# Aberrant β-Catenin Expression and Its Association With Epithelial-Mesenchymal Transition and Clinical Outcomes of Colorectal Cancer

**DOI:** 10.7759/cureus.53104

**Published:** 2024-01-28

**Authors:** Zihel H Hussein, Bashar Al Hassawi, Qais Ibraheem

**Affiliations:** 1 Department of Anatomy, Biology, and Histology, College of Medicine, University of Duhok, Duhok, IRQ

**Keywords:** mutation, apc, epithelial mesenchymal transition, β-catenin, colorectal cancer

## Abstract

Background

Colorectal cancer (CRC) is a significant global health challenge with high mortality rates. Dysregulation of β-catenin, epithelial-mesenchymal transition (EMT), and adenomatous polyposis coli (APC) are crucial in CRC development. Mutations in the APC gene lead to aberrant β-catenin expression, a key player in CRC pathogenesis. β-catenin not only influences canonical Wnt signaling but also regulates EMT. This study investigated the correlation between APC mutations, β-catenin dysregulation, and EMT induction in CRC.

Methodology

Tissue samples from 96 CRC patients and 40 para-cancerous normal tissues were collected and subjected to immunohistochemistry to assess β-catenin, E-cadherin, ZEB1, Snail, and vimentin expression. Genomic DNA was extracted and analyzed for APC mutations. Next-generation sequencing was employed for data analysis.

Results

Aberrant β-catenin expression was found in 82.3% of CRC cases and correlated with advanced clinicopathological factors. Aberrant β-catenin expression was associated with age (p=0.01), tumor invasion depth (p=0.03), nodal/distant metastasis (p=0.001 and 0.004), and vascular invasion (p=0.001). Aberrant β-catenin was correlated with EMT status. A positive correlation was observed between aberrant β-catenin expression and ZEB1 (p=0.001), Snail (p=0.001), vimentin (p=0.001), and loss of membranous E-cadherin (p=0001). Coexistence of aberrant β-catenin and EMT markers was associated with advanced CRC progression. Cancerous tissues displayed higher aberrant β-catenin and EMT markers expression than para-cancerous tissues. APC mutations were present in 59.3% of cases, with 91.2% of mutated APC cases showing aberrant β-catenin expression. The coexistence of APC mutation and aberrant β-catenin expression was correlated with the clinical outcomes of CRC patients. Mutated APC cases exhibited significantly increased EMT marker expression.

Conclusion

This study underscores the importance of aberrant β-catenin expression in CRC progression, linked to APC mutations and EMT induction. Understanding these relationships could aid in developing targeted therapies for CRC.

## Introduction

Colorectal cancer (CRC) remains a formidable global health challenge, accounting for a significant number of cancer-related deaths worldwide. Understanding the intricate molecular mechanisms governing CRC development is crucial to advance early diagnosis, treatment, and prevention strategies. Among the key molecular players that drive colorectal tumorigenesis dysregulation of β-catenin signaling, epithelial-mesenchymal transition (EMT) and adenomatous polyposis coli (APC), have emerged as focal points of research due to their pivotal roles in this malignancy [1]. β-catenin, a multifunctional protein encoded by the CTNNB1 gene, plays a central role in a multitude of cellular processes. One of its primary functions is its participation in the canonical Wnt signaling pathway, where it acts as a key regulator. In the absence of Wnt ligands, a destruction complex comprised of APC, Axin, GSK-3β, and Casein Kinase 1α (CK1α) promotes the phosphorylation and subsequent proteasomal degradation of β-catenin. This ensures low intracellular levels of β-catenin and inhibits its translocation to the nucleus. However, the onset of CRC often involves genetic mutations, particularly in the APC gene, which disrupt this regulatory mechanism. Mutations in APC lead to the accumulation of cytoplasmic β-catenin, preventing its degradation and promoting its nuclear translocation. Once in the nucleus, β-catenin forms complexes with T-cell factor/lymphoid enhancer-binding factor transcription factors, driving the transcription of oncogenic genes that are pivotal for CRC development [2].

In addition to the role of β-catenin in canonical Wnt signaling, emerging evidence has highlighted its involvement in the regulation of EMT. EMT is a fundamental biological process during embryonic development and tissue repair, where epithelial cells undergo a phenotypic switch to acquire mesenchymal characteristics. In the context of cancer, EMT confers tumor cells with enhanced migratory and invasive capabilities, facilitating their dissemination to distant sites and initiating the metastatic cascade [3]. During EMT, epithelial cells lose their cell-cell adhesion and polarity, downregulate epithelial markers (e.g., E-cadherin), and upregulate mesenchymal markers (e.g., N-cadherin, vimentin, and fibronectin). Notably, β-catenin, through its interactions with E-cadherin and transcription factors, serves as a key regulator of EMT by modulating the expression of critical EMT-inducing genes. However, investigating the potential correlation between aberrant localization of β-catenin and EMT status within CRC tissues offers a window into the underlying mechanisms of tumor development and metastasis. In this paper, we aim to comprehensively explore the intricate relationship between APC mutations, β-catenin dysregulation, and EMT induction in CRC development [4].

## Materials and methods

Tissue samples

The cross-sectional study involved 96 individuals diagnosed with CRC within the population of Duhok in Iraq, who underwent surgery at the Surgery Department of Vajeen Hospital between 2019 and 2021. After obtaining informed consent and approval from the Scientific Research Division, Duhok Directorate General of Health, Ministry of Health (13072021-7-18), tissue specimens from these patients were collected and preserved in 10% neutral buffered formalin. Subsequently, the tissues were embedded in paraffin blocks to facilitate further analysis. Additionally, 40 para-cancerous matched normal tissues were collected from the same patients for comparative studies.

Immunohistochemistry

Immunohistochemical staining was performed on 4-μm-thick tissue sections derived from the paraffin-embedded blocks. The sections were initially de-paraffinized using xylene and subsequently rehydrated through a series of ethanol concentrations. For antigen retrieval, a 0.01 M citrate buffer with a pH of 6.0 was employed in a microwave-induced process. To inhibit endogenous peroxidase activity, the sections were treated with a solution of 0.3% hydrogen peroxide in methanol for 15 minutes. The sections were exposed to the following primary antibodies at room temperature with the specified dilutions: anti- β-catenin (1:200 dilution, Dako, Glostrup, Denmark), anti-E-cadherin (1:100 dilution, Dako, Glostrup, Denmark), anti-ZEB1 (1:150 dilution, Abcam, Cambridge, United Kingdom), anti-Snail (1:500 dilution, GeneTex, California, United States), and vimentin (1:100 dilution, Dako, Glostrup, Denmark). The DAKO Kit system (Dako, Glostrup, Denmark) and a Peroxidase/DAB Kit (Dako, Glostrup, Denmark) were utilized for the immunohistochemical staining process. Subsequently, the sections were counterstained with hematoxylin, dehydrated, and finally mounted for further examination.

Immunohistochemistry analysis

Two independent pathologists utilized a semi-quantitative scoring system to evaluate ZEB1, Snail, and vimentin protein expression, taking into account the intensity of staining and the distribution of positive cells. The intensity of the positive reaction was assessed on a scale ranging from negative (0) to weak (1), moderate (2), and strong (3). Similarly, the proportion of positively stained cells was categorized into five groups: 0-5% (0), 6-25% (1), 26-50% (2), 51-75% (3), and 76-100% (4). By multiplying the staining intensity score and the proportion of positive cells, a staining index score between 0 and 12 was calculated. A staining index value of 0-6 indicated negative protein expression, whereas a score of 6-12 denoted positive protein expression [5-7]. The immunohistochemistry staining results of β-catenin and E-cadherin were assessed independently according to the subcellular localization of the nucleus, cytoplasm, and membrane. If more than 80% of cancer cells displayed membrane staining, it was considered normal expression. If more than 20% of cancer cells exhibited positive staining in the cytoplasm or nucleus, it was categorized as aberrant expression. Aberrant expression was characterized by the absence of membranous staining and the presence of ectopic expression in the cytoplasm or nucleus [8,9].

Genomic DNA extraction

Genomic DNA from formalin-fixed and paraffin-embedded-blocks specimens was isolated using a DNA extraction kit (Thermo Fisher Scientific Inc., Waltham, United States) following the manufacturer's instructions with minor modifications. Qualification and quantification of DNA concentration were performed by using NanoDrop (ND-1000, Marshall Scientific, Cambridge, United States). Samples of genomic DNA with (A260-A320)/(A280-A320) ratio of more than 1.7 and outputs more than 40 ng/μL were obtained.

Amplification and library preparation

The region between nucleotides 1 and 2883 of the APC gene was amplified using specific sets of primers overlapping exon 1-14 and exon 15 [10]. Polymerase chain reactions (PCRs) were conducted on the isolated genomic DNA samples. The resulting PCR products from each sample were combined to create PCR pools, each containing the amplicons from individual samples in a single tube. The volume of each PCR was determined based on the length of the amplicon and the efficiency of the reaction, assessed through gel electrophoresis.

The PCR pools for each sample were purified using the NucleoFast® 96 PCR kit (Macherey-Nagel GmbH & Co. KG, Düren, Germany). Subsequently, these purified pools were quantified and standardized to a concentration of 0.2 ng/μL, which was necessary for the subsequent sample preparation step. To prepare the samples for next-generation sequencing (NGS), we utilized the NexteraXT sample preparation kit (Illumina Inc., San Diego, United States), and NGS was performed using the MiSeq system (Illumina Inc., San Diego, United States). Data analysis was conducted using IGV 2.3 software (Broad Institute, Cambridge, United States). Raw reads were aligned to the hg19 reference genome using the BWA-MEM 0.7.17 tool. Subsequent steps, including sorting, duplicate marking, and base recalibration, were performed using. Variant calls were made using two separate algorithms: GATK UnifiedGenotyper and GATK HaplotypeCaller, employed in conjunction to complement each other. Low-quality variants from both sets were filtered out based on strand bias, read depth, and call quality parameters using the GATK SelectVariants option [11].

In silico analysis

Various in silico tools were harnessed to predict the impact of mutations on structural features or protein function. Polymorphism Phenotyping (PolyPhen-2) and Sorting Intolerant from Tolerant were employed to assess the functional consequences of variants. Mutation Taster was used to evaluate the effect of mutations on protein function and structure, while Align GVGD was utilized to compute a biochemical distance score.

Statistical analyses

Statistical analyses were conducted using the IBM SPSS Statistics for Windows, Version 25 (Released 2017; IBM Corp., Armonk, New York, United States). To investigate the correlations between protein expression and clinicopathological factors, as well as between cancer and normal tissues, Fisher's exact or chi-square tests were employed. All analyses were deemed statistically significant when the p-value was less than 0.05.

## Results

Clinicopathological features of colorectal cancer patients

Table 1 displays the demographic characteristics and clinicopathological features of CRC patients. Out of the 96 specimens collected, 59 patients (61.5%) were male, and 37 patients (38.5%) were female. Tumor grade demonstrated varying levels of malignancy, with the majority falling into grade II at 76%, grade III at 16.7%, and only a small portion at grade I at 7.3%. The majority of CRC patients were categorized into stage 3 (46.9%) and stage 1 (23.9%) according to tumor, node, metastasis (TNM) staging. The analysis of tumor invasion depth (T stage) showed a progressive increase in the proportion of patients, with T1 at 5.2%, T2 at 27.1%, and T3 at 53.1%. Nodal metastasis was observed in 39.6% of patients, while distant metastasis was present in only 7.3% (n=7) of cases. Notably, 60 patients (71.9%) exhibited vascular invasion, whereas perineural infiltration and lymphocytic infiltration yielded positive findings in 52.1% and 67.7% of cases, respectively.

**Table 1:** Demographic characteristics of CRC patients NO: Number; CRC: Colorectal cancer TNM staging system stands for tumor, node, metastasis. T describes the size of the tumor. N describes whether there are any cancer cells in the lymph nodes. M describes whether the cancer has spread to a different part of the body.

Variants	NO. (%)
Age	
<50	27 (28.1)
≥50	69 (71.9)
Sex	
Male	59 (61.5)
Female	37 (38.5)
Grade	
I	7 (7.3)
II	73 (76)
III	16 (16.7)
TNM Stage	
I	23 (23.9)
II	21 (21.9)
III IV	45 (46.9) 7 (7.3)
Tumor invasion	
T1	5 (5.2)
T2	26 (27.1)
T3	51 (53.1)
T4	14 (14.6)
Nodal metastasis	
N0	58 (60.4)
N1	22 (22.9)
N2	16 (16.7)
Distant metastasis	
M0	89 (92.7)
M1	7 (7.3)
Vascular invasion	69 (71.9)
Perineural invasion	50 (52.1)
Lymphocytic infiltration	65 (67.7)

Aberrant localization of β-catenin and its association with the clinical outcome of CRC patients

**Figure 1:**
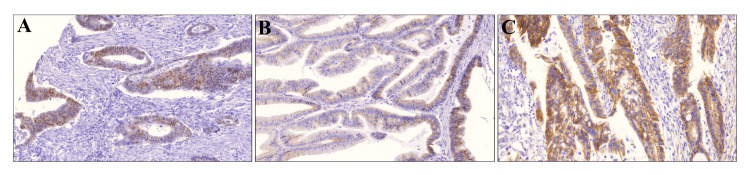
Expression and localization of β-catenin in CRC tissue specimens (A) Representative image showed intense nuclear β-catenin expression, (B) β-catenin predominantly found in the cytoplasm C. β-catenin is predominantly expressed in the cell membrane (x100). CRC: Colorectal cancer

The β-catenin protein expression and localization in CRC tissues were assessed through immunohistochemical analysis. Among the 96 samples investigated, 17 (17.7%) specimens exhibited normal β-catenin expression, located in the membrane, while most of the specimens exhibited an aberrant localization of β-catenin (79/96, 82.35%) (Table 2, Figure 1).

**Table 2:** Subcellular localization of β-catenin in CRC tissue samples CRC: Colorectal cancer

Marker	Localization	
β-catenin	Normal expression (membranous)	Aberrant expression (cytoplasm and nucleus)
	17 (17.7%)	79 (82.3%)

Additionally, the correlation between the sub-cellular localization of β-catenin and the clinicopathological features of CRC patients was investigated (Table 3). Aberrant β-catenin expression was found to be correlated with age, as cytoplasmic/nuclear β-catenin expression was more frequently detected in tissue specimens from patients over the age of 50 years old as compared to those under 50 years old (p=0.01). Moreover, the aberrant expression of β-catenin was positively associated with the depth of tumor invasion (p=0.03), number of lymph node involvement (p=0.001), distance metastasis (p=0.04), and vascular invasion (p=0.001). However, no statistically significant association was observed between the presence of aberrant β-catenin expression and gender, grade, TNM staging, perineural infiltration, and lymphocytic infiltration.

**Table 3:** Correlation between subcellular localization of β-catenin and clinicopathologic characteristics of CRC patients NO: Number; CRC: Colorectal cancer TNM staging system stands for tumor, node, metastasis. T describes the size of the tumor. N describes whether there are any cancer cells in the lymph nodes. M describes whether the cancer has spread to a different part of the body.

Variables	Total	Normal expression NO. (%)	Aberrant expression NO. (%)	p-value
Age				0.01
<50	27 (28.1)	10 (37)	17 (63)	
≥50	69 (71.9)	10 (14.5)	59 (85.5)	
Sex				0.3
Male	59 (61.5)	18 (30.5)	41 (69.5)	
Female	37 (38.5)	15 (40.5)	22 (59.5)	
Grade				0.3
I	7 (7.3)	5 (71.4)	2 (28.6)	
II	73(76)	33 (45.2)	40 (54.8)	
III	16 (16.7)	9 (56.3)	7 (43.7)	
TNM Stage				0.7
I	23(24)	14 (60.9)	9 (39.1)	
II	21(21.9)	12 (57.1)	9 (42.9)	
III	45 (46.9)	22 (48.9)	23 (51.1)	
IV	7 (7.3)	3 (42.9)	4 (57.1)	
T				0.03
T1	5 (5.2)	5 (100)	0 (0.0)	
T2	26 (27.1)	10 (38.5)	16 (61.5)	
T3	51 (53.1)	20 (39.2)	31 (60.8)	
T4	14 (14.6)	2 (14.3)	12 (85.7)	
N				0.001
N0	57 (59.4)	45 (78.9)	12 (21.1)	
N1	23 (23.9)	7 (30.4)	16 (69.6)	
N2	16 (16.7)	2 (12.5)	14 (87.5)	
M				0.04
M0	89 (92.7)	47 (52.8)	42 (47.2)	
M1	7 (7.3)	1 (14.3)	6 (85.7)	
Vascular invasion	69 (71.9)	20 (29)	49 (71)	0.001
Perineural infiltration	50 (52.1)	26 (52)	24 (48)	0.4
Lymphocytic infiltration	65 (67.7)	31 (47.7)	34 (52.3)	0.1

Combinational status of APC mutation and aberrant β-catenin expression and its association with clinicopathological features of CRC patients

**Figure 2:**
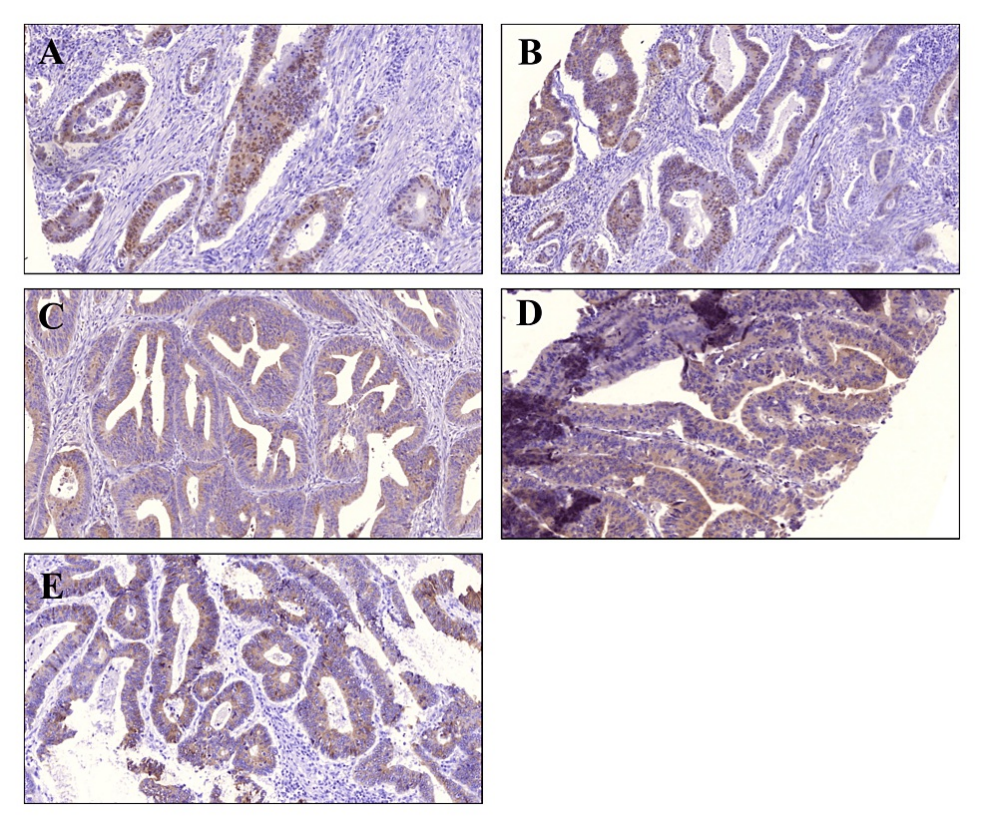
Co-expression of aberrant β-catenin and EMT-related proteins in CRC tissues Representative images from the same tumor specimens illustrate a correlation between (A) aberrant β-catenin expression and EMT markers including (B) ZEB1, (C) Snail, (D) Vimentin, and (E) Aberrant E-cadherin (x100). EMT: Epithelial-mesenchymal transition; CRC: Colorectal cancer

Our study reveals notable associations between the expression patterns of EMT markers and the aberrant localization of β-catenin (Table 4, Figure 2). We observed that positive expression of ZEB1 was significantly higher in the aberrant β-catenin expression group 82.4% (42/51) than in the normal expression group 17.6% (9/51). A similar trend was observed with Snail and vimentin. Positive expression of Snail and vimentin was detected in 70.2% (40/57) and 77.3% (37/55), whereas only 29% (17/57) and 22.7% (18/55), respectively, in cases with normal expression. Importantly, statistical analyses underscored the strong positive correlations between the expressions of these mesenchymal markers and aberrant β-catenin, with p-values of 0.001, 0.03, and 0.0001, respectively.

**Table 4:** Correlation between localization and expression of β-catenin and EMT-related proteins NO: Number; EMT: Epithelial-mesenchymal transition

Markers	Total	β-catenin		
		Normal expression NO. (%)	Aberrant expression NO. (%)	p-value
ZEB1				0.001
Positive	51 (53.1)	9 (17.6)	42 (82.4)	
Negative	45(46.9)	38 (84.4)	7 (15.6)	
Snail				0.03
Positive	57 (59.4)	17 (29.8)	40 (70.2)	
Negative	39(40.6)	20 (51.3)	19 (48.7)	
Vimentin				0.0001
Positive	55 (45.8)	18 (22.7)	37 (77.3)	
Negative	41 (54.2)	33 (78.8)	8 (21.2)	
E-cadherin				0.007
Aberrant	67 (69.8)	19 (28.4)	48 (71.6)	
Normal	29 (30.2)	18 (62.1)	11 (37.9)	

Conversely, our investigation revealed a distinct pattern for the epithelial marker E-cadherin. In aberrant localization of β-catenin specimens, 71.6% (48/67) of the cases lost membranous E-cadherin expression, significantly higher than normal β-catenin expression specimens 28.4% (19/67) (p=0.007).

The association between the coexistence of the aberrant β-catenin expression and EMT markers with clinicopathological factors of CRC was further analyzed (Table 5). Specifically, the aberrant localization of β-catenin alongside positive ZEB1 expression showed significant associations with age (p=0.003), grade (p=0.008), TNM staging (p=0.01), depth of invasion (p=0.01), nodal metastasis (p=0.01), distant metastasis (p=0.01), and vascular invasion (p=0.01). Similar findings were observed with Snail, where the coexistence of the aberrant β-catenin expression and Snail showed a significant correlation with age (p=0.08), TNM staging (p=0.003), depth of invasion (p=0.003), distant metastasis (p=0.01), vascular invasion (p=0.04), pretuneal invasion (p=0.001), and lymphocytic infiltration (p=0.02). Additionally, a comparable outcome was observed in the case of vimentin, where the coexistence of vimentin and aberrant β-catenin showed a strong correlation with vascular invasion (p=0.002), perineural invasion (p=0.004) and lymphocytic infiltration (p=0.003).

**Table 5:** Correlation between the coexistence of aberrant β-catenin and EMT-related protein expression and clinicopathologic characteristics No: Number; β-cat: β-catenin; E-cad: E-cadherin; EMT: Epithelial-mesenchymal transition The TNM staging system stands for tumor, node, metastasis. T describes the size of the tumor. N describes whether there are any cancer cells in the lymph nodes. M describes whether the cancer has spread to a different part of the body.

Variants	Positive ZEB1 No=51	β-cat/ZEB1 Positive 42 (82.4%)	p-value	Positive Snail No=57	β-cat/Snail Positive 40 (70.2%)	p-value	Positive Vimentin No=55	β-cat/Vim Positive 37 (67.3%)	p-value	Positive E-cad No=67	β-cat/E- Cad Positive 48 (71.6%)	p-value
Age	8	4 (50)	0.003	11	8 (72.7)	0.08	13	6 (46.1)	0.2	19	7 (36.8)	0.06
<50												
≥50	43	38 (88.4)		46	32 (69.6)		42	31 (73.8)		48	43 (89.6)	
Sex	32	28 (87.5)	0.4	40	27 (67.5)	0.3	37	26 (70.3)	0.8	47	40(85.1)	0.1
Male												
Female	19	14 (73.7)		17	13 (76.7)		18	11 (61.1)		20	8 (40)	
Grade	1	0 (0.0)	0.008	2	1 (50)	0.2	1	1 (100)	0.9	6	2 (33.3)	0.6
I												
II	36	30 (83.3)		42	30 (71.4)		41	28 (68.3)		50	38 (76)	
III	14	12 (85.7)		13	9 (64.3)		13	8 (61.5)		11	8 (72.7)	
TNM Stage												
I	5	1 (20)	0.01	3	1 (33.3)	0.03	2	0 (0.0)	0.4	6	2 (33.3)	0.3
II	15	12 (80)		14	9 (64.3)		27	9 (33.3)		18	7 (38.9)	
III	25	23 (92)		35	26 (74.3)		21	16 (76.2)		38	35 (92.1)	
IV	6	6 (100)		5	4 (80)		7	5 (71.4)		5	4 (80)	
T	1	0 (0.0)	0.001	1	0 (0.0)	0.003	1	0 (0.0)	0.9	2	0 (0.0)	0.8
T1												
T2	14	8 (57.1)		9	4 (44.4)		15	9 (60)		20	1 (5)	
T3	24	22 (91.7)		36	28 (77.8)		27	18 (66.7)		41	32 (78)	
T4	13	12 (92.3)		11	8 (72.7)		12	10 (83.3)		4	4 (100)	
N	17	12 (70.6)	0.001	26	15 (57.7)	0.05	26	14 (53.8)	0.8	36	22(61.1)	0.7
N0												
N1	21	17 (80.9)		20	15 (75)		15	13 (86.7)		15	12 (80)	
N2	13	13 (100)		11	10 (90.9)		11	10 (90.9)		16	14 (87.5)	
M	46	37 (80.4)	0.01	52	35 (67.3)	0.01	48	32 (66.7)	0.1	63	45(71.4)	0.9
M0												
M1	5	5 (100)		5	3 (60)		7	5 (71.4)		4	3 (75)	
Vascular invasion	45	38 (84.4)	0.01	48	39 (81.3)	0.04	53	14 (26.4)	0.002	46	41(89.1)	0.8
Perineural invasion	38	23 (60.5)	0.2	26	21 (80.8)	0.001	32	12 (37.5)	0.004	34	16 (47.1)	0.04
Lymphocytic infiltration	38	30 (78.9)	0.05	43	31 (72.1)	0.02	51	16 (31.4)	0.003	45	29 (64.4)	0.008

Concerning E-cadherin, the coexistence of aberrant localization of β-catenin and loss of membranous E-cadherin was significantly correlated with grade (p=0.01), higher lymph node involvement (p=0.001), vascular invasion (p=0.001) and lymphocytic infiltration (p=0.001). Our findings suggest that the shifting of β-catenin between its membranous to cytoplasmic/nucleus locations is associated with the initiation of EMT, and this localization pattern is also connected to the advanced progression of CRC.

Differences in expression and localization of β-catenin and EMT markers between CRC cancerous and para-cancerous tissues

This study further explores the evaluation of the subcellular localization of β-catenin, correlating this observation with the comparative analysis of EMT status between cancerous and para-cancerous matched normal tissues (Table 6). The aberrant β-catenin and E-cadherin expression were significantly frequent in cancerous tissues (p=0.001). Conversely, the normal β-catenin and E-cadherin expression were notably lower in cancerous tissues compared to their counterparts in para-cancerous matched normal tissues (p=0.001). Consistent with the findings related to aberrant β-catenin expression, the expression of mesenchymal markers, including ZEB1, Snail, and vimentin was significantly more frequent than that of normal tissues. Statistical analysis revealed a highly significant difference, with p-values of 0.004 for ZEB1, 0.0001 for Snail, and 0.007 for vimentin. Based on these findings, we observed that aberrant β-catenin expression is more common in cancerous tissues compared to controls, and this trend aligns with the presence of mesenchymal markers.

**Table 6:** Differences in expression and localization of β-catenin and EMT proteins between cancerous and cancerous-adjacent normal tissues NO: Number; EMT: Epithelial-mesenchymal transition

Markers	Cases NO. (%)	Controls NO. (%)	p-value
β-catenin			0.0001
Normal	17 (52.1)	33 (82.5)	
Aberrant	79 (47.9)	7 (17.5)	
ZEB1			0.004
Positive	51 (26)	6 (85)	
Negative	45 (74)	34 (15)	
Snail			0.0001
Positive	57 (55.2)	5 (12.5)	
Negative	39 (44.8)	35 (87.5)	
Vimentin			0.0001
Positive	55 (57.3)	2 (5)	
Negative	41 (42.7)	38 (95)	
E-cadherin			0.0001
Normal	29 (69.8)	40 (100)	
Aberrant	67 (30.2)	0 (0)	

Combinational status of APC mutation and aberrant β-catenin expression and its association with clinicopathological features of CRC patients

In our study, we conducted a mutation analysis of the APC gene using direct sequencing methods to investigate the impact of APC mutations on the subcellular localization of β-catenin and clinical outcomes in CRC patients. Out of 96 cases of CRC, 57 (59.3%) exhibited mutations within the APC gene. Among 57 mutated tumors, 31 (54.3%) and 19 (33.3%) were frameshift and non-sense mutations resulting in truncated proteins. Additionally, five cases (8.7%) exhibited point mutations leading to changes in amino acids, while two cases (3.5%) revealed silent mutations with no impact on protein structure. Remarkably, the majority of these mutations 51 out of 57 (89.4%), were concentrated within exon 15, spanning from codon 1003 to 1553, which corresponds to the β-catenin binding domain (Table 7).

**Table 7:** Sequences analysis of APC gene in CRC patients NO: Number; CRC: Colorectal cancer; APC: Adenomatous polyposis coli

Patient NO.	Codon (Exon)	Nucleotide change	Type of mutation
1	169 (4)	ATGA deletion	Frameshift
2	208 (5)	A→G	Amino acid change
3	232 (6)	C→T (Arg→stop)	Non-sense
4	367 (9)	CT deletion	Frameshift
5	430 (9)	C insertion	Frameshift
6	876 (14)	C→A (Pro→Pro)	Non-sense
7	1003 (15)	G→A (Trp→stop)	Non-sense
8	1061 (15)	AA deletion	Frameshift
9	1060 (15)	AA deletion	Frameshift
10	1114 (15)	AA deletion	Frameshift
11	1277 (15)	T→A (Leu→stop)	Non-sense
12	1286 (15)	G insertion	Frameshift
13	1282 (15)	C→G (Ser→stop)	Non-sense
14	1277 (15)	T→A (Leu→stop)	Non-sense
15	1286 (15)	G insertion	Frameshift
16	1282 (15)	C→G (Ser→stop)	Non-sense
17	1277 (15)	T→A (Leu→stop)	Non-sense
18	1309 (15)	AAA deletion	Frameshift
19	1324 (15)	G→T (Pro→Pro)	Silent
20	1470 (15)	G→T (Glu→stop)	Non-sense
21	1309 (15)	G→T (Glu→stop)	Non-sense
22	1327(15)	CGAA deletion	Frameshift
23	1282 (15)	C→G (Ser→stop)	Non-sense
24	1317 (15)	GGAA deletion	Frameshift
25	1384 (15)	G deletion	Frameshift
26	1453 (15)	T insertion	Frameshift
27	1416 (15)	G→A (Glu→Lys)	Amino acid change
28	1294 (15)	A→G (Lys→Lys)	Silent
29	1444 (15)	GG insertion	Frameshift
30	1453 (15)	G→T (Glu→stop)	Non-sense
31	1385 (15)	TA deletion	Frameshift
32	1340 (15)	CC deletion	Frameshift
33	1383 (15)	AT deletion	Frameshift
34	1277 (15)	T→A (Leu→stop)	Non-sense
35	1499 (15)	A→G (Lys→stop)	Non-sense
36	1309 (15)	G insertion	Frameshift
37	1470 (15)	G→T (Glu→stop)	Non-sense
38	1489 (15)	T→G (Leu→Val)	Amino acid change
39	14422 (15)	T insertion	Frameshift
40	1453 (15)	G→T (Glu→stop)	Non-sense
41	1378 (15)	G insertion	Frameshift
42	1493 (15)	TA deletion	Frameshift
43	1399 (15)	T→C (Leu→Pro)	Amino acid change
44	1481 (15)	TA deletion	Frameshift
45	1318 (15)	GA deletion	Frameshift
46	1309 (15)	C→T (Glu→stop)	Non-sense
47	1282 (15)	C→G (Ser→stop)	Non-sense
48	1454 (15)	T deletion	Frameshift
49	1542 (15)	G insertion	Frameshift
50	1453 (15)	G→T (Glu→stop)	Non-sense
51	1414 (15)	G→A (Gly→Glu)	Amino acid change
52	1313 (15)	C insertion	Frameshift
53	1510 (15)	G deletion	Frameshift
54	1329 (15)	A insertion	Frameshift
55	1282 (15)	G insertion	Frameshift
56	1556 (15)	A insertion	Frameshift
57	1523 (15)	GCTT insertion	Frameshift

Among the 57 cases with mutated APC, 52 cases (91.2%) exhibited aberrant β-catenin expression, while only 5 (8.8%) had normal β-catenin expression. Among the 39 patients with wild-type APC, 27 cases (69.2%) showed aberrant β-catenin expression, and 12 (30.8%) cases had normal β-catenin expression. This suggests that there is a significant association between APC mutation and aberrant β-catenin expression (p=0.005) (Table 8).

**Table 8:** Correlation between APC mutation and expression pattern of β-catenin MT: Mutated; WT: Wild type; APC: Adenomatous polyposis coli

β-catenin	APC mutation		p-value
	APC (MT)	APC (WT)	
Aberrant β-catenin	52 (91.2%)	27 (69.2%)	0.005
Normal β-catenin	5 (8.8%)	12 (30.8%)	
Total	100%	100%	

The association between the coexistence of the APC mutation and aberrant β-catenin expression and the clinicopathological factors of CRC was further analyzed (Table 9). The data suggests that the prevalence of these two markers is significantly higher in advanced stages of CRC when both molecular markers are considered simultaneously.

**Table 9:** Correlation between coexistence of APC mutation/aberrant b-catenin and clinicopathologic characteristics of CRC patients MT: Mutated; WT: Wild type; APC: Adenomatous polyposis coli; CRC: Colorectal cancer; TNM: Tumor, node, metastasis

Variables	Total				
		APC MT/aberrant β-catenin (%)	APC MT/normal β-catenin (%)	APC WT/aberrant β-catenin (%)	APC WT/normal β-catenin (%)
Age					
<50	27	6 (22.2)	2 (7.5)	11 (40.7)	8 (29.6)
≥50	69	42 (60.9)	7 (10.1)	17 (24.6)	3 (4.3)
Sex					
Male	59	38(64.4)	1(1.7)	3(5.1)	17(28.8)
Female	37	18(48.6)	0(0.0)	4(10.8)	15(40.5)
Grade					
I	7	1 (14.3)	0 (0.0)	1 (14.3)	5 (71.4)
II	73	36 (49.3)	14 (19.2)	4 (5.5)	19 (26)
III	16	6 (37.5)	0 (0.0)	1 (6.3)	9 (56.2)
TNM Stage					
I	23	2 (8.7)	1 (4.3)	7 (30.4)	13 (56.5)
II	21	5 (23.8)	3 (14.3)	4 (19)	9 (42.9)
III	45	21 (46.7)	19 (42.2)	2 (4.%)	3 (6.7)
1V	7	4 (57.1)	3 (42.9)	0 (0.0)	0 (0.0)
T					
T1	5	0 (0.0)	0 (0.0)	5 (100)	0 (0.0)
T2	26	7 (26.9)	3 (11.5)	9 (34.6)	7(26.9)
T3	51	25 (49)	8 (15.7)	6 (11.8)	12 (23.5)
T4	14	13 (92.9)	1 (7.1)	0 (0.0)	0 (0.0)
N					
N0	58	5 (8.6)	21 (36.2)	7 (12.8)	25 (43.1)
N1	22	10 (45.5)	5 (22.7)	6 (27.3)	1 (4.5)
N2	16	14 (87.5)	2 (12.5)	0 (0.0)	0 (0.0)
M					
M0	89	20 (22.5)	21 (23.6)	1 (1.1)	47 (79.7)
M1	7	6 (85.7)	1 (14.3)	0 (0.0)	0 (0.0)
Vascular invasion	69	46 (47.9)	11 (11.5)	3 (3.1)	36 (37.5)
Perineural infiltration	50	21 (21.9)	36 (37.5)	3`(3.1)	36 (37.5)
Lymphocytic infiltration	65	32 (33.3%)	25 (26.1)	2 (2.1)	37 (38.5)

APC mutation and its association with enhanced EMT markers

The connection between APC mutations and the expression of markers associated with the EMT was explored. Our findings demonstrated a noteworthy relationship between APC mutation and EMT markers (Table 10). Among the 57 cases with mutated APC, 48 cases (84.3%) were positive for ZEB1 expression, while only 9 (15.7%) had negative ZEB1 expression. Among the 39 patients with wild-type APC, only 3 cases (7.7%) showed positive ZEB1 expression, and 36 (92.3%) cases had negative ZEB1 expression. Similarly, the Snail and vimentin-positive group was more prevalent in the APC-mutated cohort (78.9 and 57.9%) compared to the wild-type APC group (30.7% and 56.4%, respectively). Regarding the epithelial marker E-cadherin, we observed a loss of membranous E-cadherin expression in 47/57 (82.4%) of mutated APC cases, compared to 20/39 (51.3%) of cases with wild-type APC. Our findings indicate a strong correlation between APC mutation and EMT, with APC-mutated samples consistently showing significantly increased expression of ZEB1, Snail, vimentin, and aberrant E-cadherin compared to cases with wild-type APC (p=0.0001, p=0.0001, p=0.04, and p=0.001, respectively).

**Table 10:** Correlation between APC mutation and expression of EMT markers MT: Mutated WT: Wild type; EMT: Epithelial-mesenchymal transition; APC: Adenomatous polyposis coli

Markers	APC mutation		p-value
	APC MT (%)	APC WT (%)	
ZEB1 positive	48 (84.3)	3 (7.7)	0.0001
ZEB1 negative	9 (15.7%)	36 (92.3)	
Snail positive	45 (78.9)	12 (30.7)	0.0001
Snail negative	12 (21.1)	27 (69.3)	
Vimentin positive	33 (57.9)	22 (56.4)	0.9
Vimentin negative	24 (42.1)	17 (43.6)	
Aberrant E-cadherin	47 (82.4)	20 (51.3)	0.001
Normal E-cadherin	10 (17.6)	19 (48.7)	

## Discussion

The aberrant localization of β-catenin within tumor cells has emerged as a significant focus of investigation in CRC due to its potential implications for disease progression and clinical outcomes. In particular, nuclear β-catenin is known to function as a transcriptional coactivator, driving the expression of genes associated with cell proliferation, survival, and tumorigenesis [12]. In our study, we conducted immunohistochemical analysis to assess the expression levels and subcellular localization of β-catenin in CRC tumor samples. Our findings demonstrate that a substantial proportion of CRC tumor samples exhibited aberrant β-catenin expression, (82.3%), while a smaller subset of samples displayed membranous β-catenin expression (11.4%). This observation is consistent with previous studies that have highlighted the prominent role of nuclear β-catenin in CRC [13]. The observed diversity in β-catenin localization suggests heterogeneity within CRC tumors, possibly reflecting different stages of tumor progression and distinct biological behaviors.

In our current study, we observed nuclear β-catenin expression or accumulation in 48% of the cancer samples, which is consistent with findings from several other studies [14,15], and this aligns with earlier studies that have documented altered β-catenin expression patterns in cancer cells. For instance, Wang and colleagues reported the absence of nuclear β-catenin accumulation in normal tissues, while it was detected in 8% of polyps, 92% of adenomas, and 100% of carcinomas [16].

The correlation between β-catenin's distinct expression patterns and clinicopathological features was also conducted. Our results reflect those of Bruun et al. and Gao et al., who also found a strong correlation between aberrant β-catenin expression and several clinicopathological characteristics [17,18]. In contrast, membranous β-catenin expression did not show a significant correlation with clinicopathological features. β-catenin, when found on the cell membrane, forms strong bonds with classical cadherins, controlling both cell-to-cell adhesion and cellular growth, ultimately inhibiting tumor progression [19].

The translocation of β-catenin from the cell membrane to the cytoplasm or nucleus can initiate the activation of EMT-related proteins and pre-invasive factors [20]. Despite extensive research into the influence of β-catenin signaling on promoting EMT in both normal and pathological contexts, this present study is the first to provide evidence for the capacity of aberrantly localized β-catenin to trigger EMT in CRC tissue specimens.

One of the most noteworthy observations from this study is the strong correlation between aberrant localization of β-catenin and the rise in mesenchymal markers; ZEB1 (p=0.001), Snail (p=0.003), and vimentin (p=0.007) coupled with the loss of membranous epithelial markers E-cadherin (p=0.001).

Furthermore, we observed that cancerous tissues exhibited increased expression of aberrant β-catenin (p=0.001) and mesenchymal markers such as ZEB1 (p<0.001), Snail (p<0.001) and vimentin (p<0.001) in comparison to normal tissues. This upregulation of protein expression followed a consistent trend, with elevated levels of β-catenin in the cytoplasm/nucleus aligning with the higher levels of mesenchymal markers typically seen in cancerous tissues.

Conversely, when we compared normal and cancerous tissues, we noted that both membranous β-catenin (p=0.001) and E-cadherin (p=0.001) protein levels were higher in normal tissues than in cancerous ones. In this context, it's important to understand the crucial role of the E-cadherin and β-catenin complexes in holding cells together. β-catenin directly binds to both the cytoplasmic domain of E-cadherin and the actin microfilament network in the cell's cytoskeleton. This binding is essential for strong cell-to-cell adhesion. It's reasonable to assume that any changes in β-catenin expression, such as its relocation from the cell membrane to the cytoplasm and nucleus, could compromise the integrity of the E-cadherin/β-catenin complex, leading to weaker cell-to-cell adhesion in cancer cells [21].

These results are consistent with existing research indicating that the translocation of β-catenin from the membrane to the nucleus is associated with the disruption of epithelial characteristics and the acquisition of a mesenchymal phenotype highlighting the pivotal role of β-catenin in EMT-related changes in cell adhesion and morphology [22].

Importantly, the study also links these molecular observations to clinical factors in CRC patients. The associations between aberrant β-catenin expression and high expression of EMT markers with clinicopathological factors such as grade, stage, depth of invasion, nodal metastasis, distant metastasis, and vascular invasion underscore the clinical relevance of these molecular findings.

These associations suggest that patients with CRC displaying high aberrant β-catenin and EMT marker expression may present with a more aggressive and advanced form of the disease, the same result conducted by Son and Moon 2010 [23].

The APC gene plays a central role in the regulation of β-catenin levels through the canonical Wnt signaling pathway [24]. APC is located on chromosome 5 and consists of 21 exons [25]. Exon 15 of APC is the most important, as it comprises 75% of the coding sequence of APC and hence is the common target for both germline and somatic mutations, which usually span codons 1286-1513 of this exon. This region represents the mutation cluster region, corresponding to the b-catenin binding domain and 68-77% of somatic mutations in APC occur in this region. As APC mutations disrupt the degradation of β-catenin, leading to its accumulation in both the cytoplasm and nucleus [26], we undertook an investigation into the impact of APC mutations on the aberrant localization of β-catenin. First, in our initial screening, we investigated APC mutations within our samples; the overall mutation rate of APC among the 96 patients was 59.4%. The majority of these mutations 89.4% were concentrated within exon 15, spanning from codon 1003 to 1553, which corresponds to the β-catenin binding domain.

Additionally, 87.6% of these mutations were frameshift and non-sense mutations resulting in truncated proteins. Second, in our exploratory analysis aimed at uncovering the connection between APC mutations and aberrant β-catenin expression, our results revealed a compelling coexistence between APC mutations and aberrant nuclear β-catenin expression within the same tumor samples. Specifically, in cases where APC mutations were detected, β-catenin showed a distinct localization within the nucleus or cytoplasm of the tumor cells. These results support previous research into this brain area which links APC mutation and subcellular localization of β-catenin [27].

While previous studies have separately explored the correlation of APC mutation or aberrant β-catenin expression with the pathological features of cancer patients [28], our study marks the first investigation of the combined status of these two markers in relation to the pathological characteristics of CRC patients. We found that the coexistence of APC mutation and aberrant β-catenin was notably more prevalent in advanced stages of CRC.

Having established a link between APC mutations and the aberrant β-catenin expression, as well as a connection between the aberrant β-catenin and the status of EMT, it is justifiable to hypothesize a connection between APC mutations and EMT status. To investigate this hypothesis, we conducted an analysis, and our findings revealed a strong association between APC mutation and EMT, as indicated by the expression levels of EMT markers in two groups: those with APC mutations and those without APC mutation.

In mutated APC samples, we consistently observed a significantly higher percentage of positive expression for mesenchymal markers, such as ZEB1 and Snail compared to wild-type samples.

These results are in agreement with Sánchez-Tilló et al. 2011 findings which showed that ZEB1 is found in the epithelial cells of intestinal tumors in both human patients with familial adenomatous polyposis (APC mutations causing β-catenin nuclear accumulation) and mouse models (APCMin/+). However, it is not present in the epithelium of CRCs with wild-type APC and no nuclear β-catenin accumulation [29].

Furthermore, in cases where APC mutations were detected, there was a notable loss of E-cadherin expression from the cell membrane. The altered subcellular localization of β-catenin, coupled with the reduction of E-cadherin at the cell membrane, strongly suggests that APC mutations may lead to aberrant signaling pathways involving β-catenin and E-cadherin, which are critical components of the Wnt signaling pathway and cell adhesion, respectively [30].

Limitations and future directions

Despite the valuable insights provided by our study, there are limitations to consider. The sample size in our study was relatively small, and further research with a larger cohort is needed to validate these findings. Additionally, the molecular mechanisms underlying the regulation of β-catenin in CRC and its interplay with EMT require further investigation. This study contributes to the understanding of the complex interplay between APC mutations, β-catenin dysregulation, and EMT in CRC. These findings have implications for the development of diagnostic and therapeutic strategies for this challenging malignancy. Further research in this area is warranted to explore the full potential of targeting β-catenin signaling pathways in CRC treatment and prevention.

## Conclusions

Intriguingly, our comprehensive investigation of colon cancer cases has unveiled a multifaceted relationship among key molecular players. Notably, the coexistence of APC mutations with heightened aberrant β-catenin expression is accompanied by a concomitant upregulation of markers associated with the EMT. In these cases, where APC mutations were detected, the expression levels of mesenchymal markers such as ZEB1, Snail, and vimentin exhibited significant upregulation. This intriguing correlation between APC mutations, aberrant localization of β-catenin, and an enhanced EMT status suggests a complex interplay between these factors in colon cancer progression. It implies that the dysregulation of the Wnt/β-catenin pathway due to APC mutations might play a pivotal role not only in nuclear β-catenin accumulation but also in orchestrating the EMT process, a hallmark of tumor invasiveness and metastasis.
